# Vital Signs: Colorectal Cancer Screening Test Use — United States, 2012

**Published:** 2013-11-08

**Authors:** Carrie N. Klabunde, Djenaba A. Joseph, Jessica B. King, Arica White, Marcus Plescia

**Affiliations:** Applied Research Program, National Cancer Institute, Bethesda, Maryland; Div of Cancer Prevention and Control, National Center for Chronic Disease Prevention and Health Promotion, CDC

## Abstract

**Background:**

Strong evidence exists that screening with fecal occult blood testing (FOBT), sigmoidoscopy, or colonoscopy reduces the number of deaths from colorectal cancer (CRC). The percentage of the population up-to-date with recommended CRC screening increased from 54% in 2002 to 65% in 2010, primarily through increased use of colonoscopy.

**Methods:**

Data from the 2012 Behavioral Risk Factor Surveillance System survey were analyzed to estimate percentages of adults aged 50–75 years who reported CRC screening participation consistent with United States Preventive Services Task Force recommendations.

**Results:**

In 2012, 65.1% of U.S. adults were up-to-date with CRC screening, and 27.7% had never been screened. The proportion of respondents who had never been screened was greater among those without insurance (55.0%) and without a regular care provider (61.0%) than among those with health insurance (24.0%) and a regular care provider (23.5%). Colonoscopy was the most commonly used screening test (61.7%), followed by FOBT (10.4%). Colonoscopy was used by more than 53% of the population in every state. The percentages of blacks and whites up-to-date with CRC screening were equivalent. Compared with whites, a higher percentage of blacks across all income and education levels used FOBT.

**Conclusions:**

Many age-eligible adults did not use any type of CRC screening test as recommended. Organized, population-based approaches might increase CRC screening among those who have never been screened. Promoting both FOBT and colonoscopy as viable screening test options might increase CRC screening rates and reduce health disparities.

## Introduction

Colorectal cancer (CRC) is the second most common cause of cancer death among cancers that affect both men and women (1). Strong evidence exists that screening for CRC reduces the incidence and mortality of the disease (2). Approximately 90% of those diagnosed with early stage cancer live 5 or more years (3). Screening with either a fecal occult blood test (FOBT) or sigmoidoscopy has been shown in randomized controlled trials to decrease CRC mortality (2). Currently, no randomized controlled trials demonstrate the efficacy of colonoscopy; however, observational studies have reported a reduction in CRC incidence (2). The United States Preventive Services Task Force (USPSTF) recommends several tests for the prevention or early detection of CRC among adults ages 50–75 years: 1) high-sensitivity FOBT annually, 2) colonoscopy every 10 years, or 3) sigmoidoscopy every 5 years with FOBT every 3 years (4).

The percentage of the U.S. adult population that is up-to-date with recommended CRC screening increased from 54% in 2002 to 65% in 2010, primarily driven by increased use of colonoscopy (5). Use of FOBT and sigmoidoscopy declined steadily over the same period (5). This report describes current CRC screening test use by state and type of test, using data from the 2012 Behavioral Risk Factor Surveillance System (BRFSS) survey.

## Methods

BRFSS is an annual, state-based, random-digit-dialed telephone survey of the civilian, noninstitutionalized adult population aged ≥18 years that collects information on health risk behaviors, preventive health practices, and health-care access in the United States. Survey data were available for all 50 states and the District of Columbia (DC). The median combined response rate for the 2012 BRFSS survey was 45.2%.

BRFSS respondents aged ≥50 years were asked whether they had ever used “a special kit at home to determine whether the stool contains blood (FOBT),” whether they had ever had a “tube inserted in the rectum to view the colon for signs of cancer or other health problems (sigmoidoscopy or colonoscopy),” and if so, whether their “most recent exam was a sigmoidoscopy or a colonoscopy” and when these tests were last performed. In accordance with current USPSTF guidelines for CRC screening, the percentages of adults aged 50–75 years who reported having had a FOBT within the past year, colonoscopy within the previous 10 years, or sigmoidoscopy within the previous 5 years and FOBT within the previous 3 years were estimated as in previous reports (6). Of 236,565 respondents aged 50–75 years, a total of 15,985 (6.8%) who declined to answer, had a missing answer, or who answered “don’t know/not sure” were excluded from the analysis. Screening status (up-to-date with CRC screening, screened but not up-to-date, and never screened) was analyzed by demographic variables. The composite measure (up-to-date with CRC screening), use of colonoscopy, and use of FOBT were examined by demographic variables and by state; because of small numbers, data were not presented for sigmoidoscopy in combination with FOBT. Data were weighted to the age, sex, and racial/ethnic distribution of each state’s adult population using intercensal estimates that were age standardized to the 2012 BRFSS population.

## Results

In 2012, 65.1% of respondents reported they were up to date with one of the CRC screening tests recommended by the USPSTF (Table 1). Of respondents, 7.2% had been screened, but were not up-to-date, and 27.7% reported they had never been screened. The percentages of blacks and whites who reported being up-to-date with CRC screening were essentially equivalent and greater than those for other races. The percentages that had never been screened were highest for ages 50–64 years, men, Hispanics, American Indian/Alaska Natives and those who live in non-metropolitan areas. As education level and annual household income increased, the proportion of respondents who had never been screened decreased. The proportion of respondents who had never been screened was greater among those without insurance (55.0%) and without a regular care provider (61.0%) than among those with health insurance (24.0%) and a regular care provider (23.5%).

Among respondents who were up-to-date with CRC screening, colonoscopy was the most commonly used test (61.7%), followed by FOBT (10.4%), and sigmoidoscopy in combination with FOBT (0.7%) (Table 2). The percentage reporting use of either FOBT or colonoscopy increased with age and was greater among those with health insurance and those with a regular health-care provider. Compared with other racial groups, a greater percentage of whites (62.7%) and blacks (62.1%) reported colonoscopy within 10 years, and a greater percentage of Asian/Pacific Islanders (14.5%) and blacks (12.6%) reported FOBT within 1 year. Minimal variation in reported FOBT use by education level and household income was observed, whereas the percentage of respondents reporting colonoscopy within the last 10 years increased with greater education level and annual household income. Among blacks and whites, a greater percentage of blacks reported receiving an FOBT within 1 year regardless of income or education level (Figure).

The percentage of respondents who were up-to-date with CRC screening was highest in Massachusetts (76.3%) and lowest in Arkansas (55.7%) and Wyoming (55.9%) (Table 3). In every state, at least 53% of respondents reported receiving colonoscopy within 10 years. California had the highest percentage of respondents who reported FOBT within 1 year (20.2%) and Utah had the lowest percentage (3.4%). The percentage of respondents in any state reporting receiving sigmoidoscopy within 5 years and FOBT within 3 years was ≤3%.

## Discussion

Approximately two-thirds of the U.S. population aged 50–75 years were up-to-date with CRC screening in 2012. Previous studies suggest CRC screening rates are increasing less rapidly than in the past (6). By far the most commonly used CRC screening test was colonoscopy. Colonoscopy use was similar for whites and blacks, but varied by education and household income. A much smaller percentage of eligible adults used FOBT. FOBT use was similar by education and household income overall, but a greater percentage of blacks across all education and income levels reported use of FOBT. The percentage of the eligible population that used sigmoidoscopy with FOBT was extremely low. States with higher screening rates had higher use of FOBT and/or colonoscopy, with considerable variation by state.

Although no CRC screening strategy has been shown to be superior when the risk and benefits of each test are considered (2), this study found that colonoscopy is the predominant method for CRC screening in the United States. Primary-care providers are the most common source for a CRC screening recommendation. Many providers believe that colonoscopy is the best test option and do not offer other screening tests to their patients (7–8). Colonoscopy can detect and remove precancerous polyps during the procedure, but it is an invasive procedure and requires significant patient preparation and time commitment.

This study showed FOBT was infrequently used. Most primary-care physicians still offer FOBT (although sometimes an older, less-sensitive guaiac FOBT) to their patients at least some of the time, although most report thinking that FOBT is only somewhat effective in reducing CRC mortality (9). Newer tests, such as the high-sensitivity guaiac FOBT and high-sensitivity fecal immunochemical test (FIT) are recommended for CRC screening in current guidelines (4). FOBT is relatively inexpensive, easy to use, and widely available, but requires more frequent repeat testing with prompt subsequent colonoscopy in all those with a positive test. This study found that use of FOBT and colonoscopy varied by demographic characteristics and by state. This variation might be attributed to patient preferences, provider preferences, or other factors such as physician reimbursement policies and availability of certain tests. Patients have strong preferences for particular CRC screening tests, but many, particularly those in minority populations, would choose FOBT when provided with objective information about test options (10–12). Evidence also indicates that patients choosing FOBT are more likely to complete the test than those who choose colonoscopy (10,13).

The potential to increase screening rates exists if health-care providers identify the test that their patient is most likely to complete and consistently offer all recommended screening tests. This study found that most states with higher overall CRC screening percentages also had relatively higher use of FOBT and colonoscopy, although FOBT use was much lower than would be expected based on studies of patient preference and subsequent adherence (10–12). The study also found that blacks and whites have approximately the same screening rates, but a higher percentage of blacks across all income and education levels used FOBT.

A substantial percentage of persons who were without insurance or did not have a regular health-care provider had not been screened for CRC, and were unlikely to have had regular contact with the health care system. Although the Affordable Care Act will help address these barriers by providing coverage for CRC screening tests without additional costs, the traditional reliance on primary-care settings to promote and provide cancer screenings will only reach those who have regular contact with the health-care system (13). Additional analyses showed that among those who had never been screened, 76% actually had health insurance, so additional interventions are needed even among those with access to health care. Organized screening systems identify eligible populations, reach out to persons in their homes or community settings, and carefully monitor adherence and follow-up of abnormal tests. Such approaches have been widely applied to other clinical preventive services, such as immunization and screening for sexually transmitted diseases, and have been successful in substantially increasing CRC screening in several settings (13–16). A recent randomized controlled trial of uninsured patients who were not up-to-date with CRC screening demonstrated that mailings to patients identified as eligible for screening substantially increased CRC screening participation, with significantly higher screening rates among those sent a FIT test kit than among those offered colonoscopy (13). To accelerate progress in increasing CRC screening, public health agencies might consider supporting organized screening approaches by developing population-level interventions to improve cancer screening across communities, and using communication and outreach in communities with low CRC screening rates (17).

**Key Points**

- About 1 in 3 adults aged 50–75 years have not been screened for colorectal cancer according to national guidelines.
- Of adults who have been screened, colonoscopy is the most commonly used colorectal cancer screening test. Only 1 in 10 screened adults have used fecal occult blood tests (FOBT).
- Blacks and whites had equivalent colorectal cancer screening rates. Compared with whites, a higher percentage of blacks across all income and education levels used FOBT.
- To increase use of colorectal cancer screening tests, state and local public health can 1) work with existing programs, doctors and public health professionals who have already greatly increased colorectal cancer screening rates; 2) develop record systems to track and explore ways to increase screening rates; 3) promote all three testing options to key audiences; 4) use public health workers and patient navigators in communities with low testing rates; and 5) work with state Medicaid programs, primary-care associations, and Medicare quality improvement organizations.
- Additional information is available at http://www.cdc.gov/vitalsigns.

The findings in this report are subject to at least four limitations. First, CRC screening rates might be overestimated or underestimated because BRFSS does not specify whether testing was done for screening or diagnosis. Second, data are self-reported and not validated by medical records review. Third, response rates were low (45.2%), although the BRFSS weighting procedure corrects for nonresponse, and 6.8% of respondents did not answer all the questions and were excluded. Finally, in 2011, the sampling frame for BRFSS expanded to include cellular telephones, resulting in changes to the weighting of BRFSS data (18). Therefore, data collected before 2011 cannot be compared with or presented in trend analysis with data collected in 2011 or subsequent years.

In the U.S. population, 65.1% of adults are currently up-to-date with CRC screening recommendations based on self-reported BRFSS survey data. Progress to date has been driven almost exclusively by use of colonoscopy, which was used by more than half of the population in every state. Compared with whites, a higher percentage of blacks across all income and education levels used FOBT. CDC’s Colorectal Cancer Control Program has set a goal of increasing the CRC screening rate to 80% by 2014. To achieve this goal, aggressive approaches will be needed, including more consistent promotion of both FOBT and colonoscopy as viable screening options and development of organized, population-based strategies that extend CRC screening efforts to settings beyond the medical provider’s office.

## Figures and Tables

**FIGURE:**
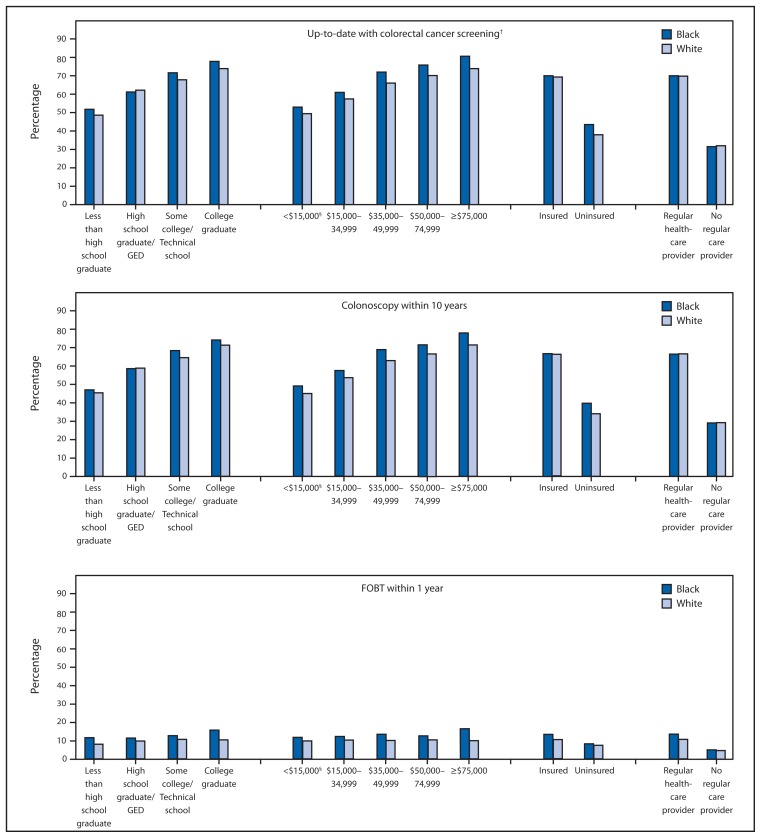
Percentage of black and white respondents aged 50–75 years who reported colorectal cancer screening test use, by test type and selected characteristics — Behavioral Risk Factor Surveillance System (BRFSS), United States, 2012^*^ **Abbreviations:** FOBT = fecal occult blood test; GED = general equivalency diploma. ^*^ Data were weighted to the age, sex, and racial/ethnic distribution of each state’s adult population using intercensal estimates and were age-standardized to the 2012 BRFSS population. ^†^ FOBT within 1 year, or sigmoidoscopy within 5 years with FOBT within 3 years, or colonoscopy within 10 years. ^§^ Annual household income.

**TABLE 1:** Percentage of respondents age 50–75 years who reported colorectal cancer (CRC) screening test use, by screening status and selected charactereristics — Behavioral Risk Factor Surveillance System (BRFSS), United States, 2012*

	Up-to-date with CRC screening†		Screened but not up-to-date		Never screened	
			
Characteristic	%	(95% CI)	%	(95% CI)	%	(95% CI)
**Overall**	**65.1**	**(64.7–65.5)**	**7.2**	**(7.0–7.5)**	**27.7**	**(27.3–28.1)**
**Age (yrs)**						
50–64	60.0	(59.5–60.5)	7.0	(6.7–7.3)	33.0	(32.5–33.5)
65–75	76.8	(76.2–77.4)	7.8	(7.5–8.1)	15.4	(14.8–15.9)
**Sex**						
Men	63.9	(63.2–64.5)	6.5	(6.2–6.9)	29.6	(29.0–30.2)
Women	66.2	(65.7–66.8)	7.9	(7.6–8.2)	25.9	(25.4–26.4)
**Race**						
White	65.9	(65.4–66.3)	7.5	(7.2–7.7)	26.7	(26.3–27.1)
Black	65.5	(64.2–66.9)	5.9	(5.3–6.6)	28.5	(27.2–29.9)
Asian/Pacific Islander	63.2	(58.9–67.2)	6.6	(4.7–9.3)	30.2	(26.4–34.3)
American Indian/Alaska Native	54.5	(50.8–58.2)	6.2	(4.9–7.7)	39.3	(35.6–43.1)
Other/Multiracial	51.2	(47.7–54.7)	6.0	(4.7–7.6)	42.9	(39.4–46.4)
**Ethnicity**						
Hispanic	53.1	(51.1–55.1)	5.9	(4.9–6.9)	41.0	(39.0–43.1)
Non-Hispanic	66.4	(66.0–66.8)	7.4	(7.2–7.6)	26.3	(25.9–26.6)
**Education**						
Less than high school graduate	48.3	(46.7–49.8)	6.6	(5.9–7.4)	45.1	(43.6–46.6)
High school graduate/GED	61.7	(60.9–62.4)	7.1	(6.7–7.4)	31.3	(30.5–32.0)
Some college/Technical school	67.8	(67.1–68.6)	7.8	(7.4–8.2)	24.4	(23.7–25.1)
College graduate	73.5	(72.8–74.1)	7.1	(6.8–7.5)	19.4	(18.8–20.0)
**Annual household income ($)**						
<15,000	49.5	(48.0–50.9)	8.0	(7.3–8.8)	42.5	(41.0–44.0)
15,000–34,999	57.1	(56.2–58.1)	8.2	(7.7–8.7)	34.7	(33.8–35.6)
35,000 –49,999	66.4	(65.3–67.5)	7.2	(6.7–7.8)	26.4	(25.4–27.5)
50,000–74,999	70.4	(69.4–71.4)	6.8	(6.3–7.3)	22.9	(21.9–23.8)
≥75,000	74.0	(73.3–74.7)	6.4	(6.0–6.9)	19.5	(18.9–20.2)
**Residence location** §						
Metropolitan	68.7	(68.1–69.3)	7.4	(7.0–7.7)	23.9	(23.3–24.5)
Non-metropolitan	64.8	(64.1–65.4)	7.3	(7.0–7.6)	28.0	(27.4–28.5)
**Health insurance status**						
Yes	68.9	(68.5–69.4)	7.1	(6.8–7.3)	24.0	(23.6–24.4)
No	36.9	(34.9–39.0)	8.0	(7.3–8.9)	55.0	(52.9–57.1)
**Regular health-care provider status**						
Yes	69.3	(68.9–69.8)	7.1	(6.9–7.4)	23.5	(23.1–23.9)
No	30.7	(29.3–32.0)	8.4	(7.7–9.1)	61.0	(59.5–62.4)

**Abbreviations:** CRC = colorectal cancer; CI = confidence interval; GED = general equivalency diploma.

* Data were weighted to the age, sex, and racial/ethnic distribution of each state’s adult population using intercensal estimates and were age-standardized to the 2012 BRFSS population.

† Fecal occult blood test (FOBT) within 1 year, or sigmoidoscopy within 5 years with FOBT within 3 years, or colonoscopy within 10 years.

§ Metropolitan is defined as in the center city of a metropolitan statistical area (MSA) or outside the center city of an MSA but not inside the county containing the center city. Non-metropolitian is defined as inside a suburban county of the MSA, in an MSA that has no center city, or not in an MSA.

**TABLE 2:** Percentage of respondents aged 50–75 years who reported colorectal cancer (CRC) screening test use, by test type and selected characteristics — Behavioral Risk Factor Surveillance System (BRFSS), United States, 2012*

	Up-to-date with CRC screening†		Colonoscopy within 10 years		FOBT within 1 year	
			
Characteristic	%	(95% CI)	%	(95% CI)	%	(95% CI)
**Overall**	**65.1**	**(64.7–65.5)**	**61.7**	**(61.2–62.1)**	**10.4**	**(10.1–10.6)**
**Age (yrs)**						
50–64	60.0	(59.5–60.5)	56.4	(55.8–56.9)	8.9	(8.6–9.3)
65–75	76.8	(76.2–77.4)	73.9	(73.2–74.5)	13.6	(13.1–14.2)
**Sex**						
Men	63.9	(63.2–64.5)	60.5	(59.8–61.1)	10.6	(10.2–11.0)
Women	66.2	(65.7–66.8)	62.8	(62.2–63.3)	10.2	(9.8–10.5)
**Race**						
White	65.9	(65.4–66.3)	62.7	(62.3–63.1)	10.0	(9.7–10.2)
Black	65.5	(64.2–66.9)	62.1	(60.6–63.5)	12.6	(11.6–13.7)
Asian/Pacific Islander	63.2	(58.9–67.2)	54.6	(50.0–59.1)	14.5	(11.5–18.0)
American Indian/Alaska Native	54.5	(50.8–58.2)	49.5	(45.8–53.3)	11.3	(9.2–13.9)
Other/Multiracial	51.2	(47.7–54.7)	49.1	(45.6–52.6)	6.9	(5.6–8.5)
**Ethnicity**						
Hispanic	53.1	(51.1–55.1)	48.4	(46.4–50.5)	10.2	(9.0–11.5)
Non-Hispanic	66.4	(66.0–66.8)	63.1	(62.7–63.5)	10.4	(10.1–10.6)
**Education level**						
Less than a high school graduate	48.3	(46.7–49.8)	44.7	(43.2–46.2)	8.4	(7.7–9.3)
High school graduate/GED	61.7	(60.9–62.4)	58.2	(57.4–59.0)	9.9	(9.5–10.4)
Some college/Technical school	67.8	(67.1–68.6)	64.2	(63.4–65.0)	11.1	(10.6–11.7)
College graduate	73.5	(72.8–74.1)	70.5	(69.8–71.2)	10.9	(10.5–11.4)
**Annual household income ($)**						
<15,000	49.5	(48.0–50.9)	45.0	(43.5–46.4)	10.2	(9.4–11.1)
15,000–34,999	57.1	(56.2–58.1)	53.1	(52.2–54.1)	10.4	(9.8–11.0)
35,000–49,999	66.4	(65.3–67.5)	63.1	(62.0–64.2)	10.5	(9.8–11.3)
50,000–74,999	70.4	(69.4–71.4)	66.8	(65.8–67.9)	10.8	(10.1–11.6)
≥75,000	74.0	(73.3–74.7)	71.3	(70.6–72.1)	10.5	(9.9–11.0)
**Residence location** §						
Metropolitan	68.7	(68.1–69.3)	64.9	(64.2–65.5)	11.7	(11.3–12.2)
Non-metropolitan	64.8	(64.1–65.4)	62.2	(61.5–62.8)	8.9	(8.5–9.2)
**Health insurance status**						
Yes	68.9	(68.5–69.4)	65.5	(65.1–66.0)	10.9	(10.6–11.2)
No	36.9	(34.9–39.0)	33.1	(31.2–35.2)	7.0	(6.0–8.1)
**Regular health-care provider status**						
Yes	69.3	(68.9–69.8)	65.9	(65.4–66.3)	11.1	(10.8–11.4)
No	30.7	(29.3–32.0)	28.0	(26.7–29.3)	4.6	(4.0–5.2)

**Abbreviations:** CI = confidence interval; FOBT = fecal occult blood test; GED = general equivalency diploma.

* Data were weighted to the age, sex, and racial/ethnic distribution of each state’s adult population using intercensal estimates and were age-standardized to the 2012 BRFSS population.

† FOBT within 1 year, or sigmoidoscopy within 5 years with FOBT within 3 years, or colonoscopy within 10 years.

§ Metropolitan is defined as in the center city of a metropolitan statistical area (MSA) or outside the center city of an MSA but not inside the county containing the center city. Non-metropolitian is defined as inside a suburban county of the MSA, in an MSA that has no center city, or not in an MSA.

**TABLE 3:** Percentage of respondents aged 50–75 years who reported colorectal cancer (CRC)screening test use, by test type and by state ranked by percentage who were up-to-date with CRC screening — Behavioral Risk Factor Surveillance System, United States, 2012*

State	Up–to–date with CRC screening†		Colonoscopy within 10 years		FOBT within 1 year	
		
	%	(95% CI)	%	(95% CI)	%	(95% CI)
**Overall**	**65.1**	**(64.7–65.5)**	**61.7**	**(61.2–62.1)**	**10.4**	**(10.1–10.6)**
**Highest tertile**						
Massachusetts	76.3	(74.9–77.6)	73.7	(72.3–75.1)	9.9	(9.0–10.8)
New Hampshire	75.3	(73.4–77.0)	73.6	(71.7–75.4)	7.8	(6.8–8.8)
Maine	73.1	(71.6–74.6)	71.1	(69.6–72.6)	8.4	(7.6–9.3)
Rhode Island	72.7	(70.5–74.9)	71.0	(68.7–73.1)	8.1	(6.9–9.4)
Connecticut	72.1	(70.1–74.0)	69.9	(67.9–71.8)	10.4	(9.3–11.7)
Vermont	71.4	(69.4–73.3)	69.5	(67.5–71.5)	7.8	(6.8–9.0)
Delaware	71.2	(68.6–73.6)	70.0	(67.4–72.5)	7.1	(6.0–8.4)
Wisconsin	71.2	(68.4–73.7)	69.1	(66.4–71.7)	6.3	(5.1–7.6)
Minnesota	70.6	(69.0–72.1)	69.5	(67.9–71.1)	4.7	(4.1–5.4)
Maryland	70.4	(68.6–72.2)	68.1	(66.2–69.9)	11.4	(10.3–12.6)
New York	69.4	(66.8–71.9)	67.0	(64.3–69.6)	8.2	(6.9–9.8)
Michigan	69.0	(67.3–70.7)	67.4	(65.7–69.1)	9.4	(8.4–10.4)
North Carolina	68.2	(66.5–69.8)	65.1	(63.4–66.7)	11.0	(10.0–12.1)
Virginia	68.0	(66.0–69.9)	65.8	(63.8–67.8)	9.5	(8.4–10.7)
Utah	68.0	(66.3–69.6)	67.1	(65.4–68.7)	3.4	(2.9–4.1)
Georgia	67.2	(64.9–69.5)	64.4	(62.1–66.7)	11.8	(10.3–13.4)
California	67.1	(65.2–68.8)	57.3	(55.3–59.2)	20.2	(18.8–21.8)
**Middle tertile**						
Washington	66.8	(65.4–68.2)	63.8	(62.4–65.3)	10.1	(9.3–10.9)
District of Columbia	66.7	(62.9–70.3)	63.4	(59.6–67.0)	14.1	(12.1–16.3)
Pennsylvania	66.5	(65.1–68.0)	63.6	(62.1–65.1)	9.0	(8.1–9.9)
Iowa	65.9	(64.0–67.7)	63.9	(62.0–65.7)	8.6	(7.6–9.7)
Colorado	65.4	(63.8–66.9)	61.3	(59.7–62.9)	10.1	(9.1–11.2)
Alabama	64.9	(63.0–66.8)	62.4	(60.4–64.3)	9.5	(8.5–10.6)
Oregon	64.7	(62.3–67.0)	61.3	(58.8–63.7)	9.8	(8.4–11.4)
Kansas	64.6	(63.0–66.1)	61.4	(59.8–62.9)	10.7	(9.8–11.8)
Tennessee	64.3§	(62.1–66.5)	62.2	(59.9–64.3)	10.2	(9.0–11.5)
Florida	64.2	(61.8–66.5)	60.9	(58.4–63.3)	12.5	(11.0–14.1)
South Carolina	64.2	(62.4–65.9)	62.6	(60.8–64.4)	6.9	(6.2–7.8)
Hawaii	64.1	(61.6–66.6)	56.5	(53.8–59.1)	14.6	(12.9–16.4)
Missouri	64.0	(61.6–66.3)	61.0	(58.5–63.4)	7.6	(6.5–8.9)
Ohio	63.3	(61.7–64.9)	59.7	(58.0–61.3)	9.2	(8.3–10.2)
Kentucky	62.9	(61.0–64.8)	60.2	(58.2–62.1)	8.6	(7.6–9.8)
West Virginia	62.7	(60.6–64.8)	59.0	(56.8–61.1)	12.7	(11.4–14.1)
New Jersey	62.4	(60.6–64.0)	60.1	(58.3–61.8)	7.8	(7.0–8.7)
**Lowest tertile**						
South Dakota	62.3	(59.6–65.0)	59.8	(57.0–62.5)	8.5	(7.1–10.1)
Illinois	61.3	(58.8–63.8)	59.4	(56.9–61.9)	6.0	(5.0–7.2)
Nebraska	60.9	(59.5–62.3)	58.2	(56.8–59.7)	7.3	(6.6–8.1)
Indiana	60.2	(58.2–62.0)	57.3	(55.4–59.2)	8.8	(7.8–10.0)
Idaho	59.8	(56.8–62.6)	58.0	(55.1–60.8)	7.2	(5.9–8.7)
Louisiana	59.8	(57.7–61.9)	56.2	(54.1–58.3)	10.7	(9.5–12.1)
Texas	58.5	(56.3–60.7)	55.7	(53.5–57.9)	8.6	(7.4–10.0)
Oklahoma	58.3	(56.4–60.1)	55.1	(53.2–57.0)	8.0	(7.0–9.0)
Arizona	58.0	(55.2–60.6)	55.2	(52.5–57.9)	9.4	(8.0–11.0)
Mississippi	58.0	(56.0–60.0)	55.0	(53.0–57.1)	11.1	(9.9–12.4)
Nevada	58.0	(54.8–61.3)	54.4	(51.1–57.6)	11.4	(9.5–13.7)
North Dakota	57.9	(55.5–60.4)	54.9	(52.5–57.4)	8.1	(6.9–9.5)
Alaska	57.6	(54.6–60.7)	54.8	(51.7–57.9)	7.3	(5.8–9.2)
New Mexico	57.5	(55.6–59.3)	54.4	(52.5–56.2)	8.6	(7.5–9.7)
Montana	56.2	(54.3–58.1)	53.4	(51.4–55.3)	6.5	(5.6–7.5)
Wyoming	55.9	(53.3–58.4)	53.7	(51.1–56.2)	5.4	(4.4–6.5)
Arkansas	55.7	(53.2–58.2)	53.4	(50.8–55.9)	8.3	(7.0–9.8)

**Abbreviations:** CI = confidence interval; FOBT = fecal occult blood test.

* Data were weighted to the age, sex, and racial/ethnic distribution of each state’s adult population using intercensal estimates and were age–standardized to the 2012 BRFSS population.

† FOBT within 1 year, or sigmoidoscopy within 5 years with FOBT within 3 years, or colonoscopy within 10 years.

§ Median.
